# Mapping of agronomic traits, disease resistance and malting quality in a wide cross of two-row barley cultivars

**DOI:** 10.1371/journal.pone.0219042

**Published:** 2019-07-17

**Authors:** Rachel Goddard, Sarah de Vos, Andrew Steed, Amal Muhammed, Keith Thomas, David Griggs, Christopher Ridout, Paul Nicholson

**Affiliations:** 1 Department of Crop Genetics, John Innes Centre, Norwich Research Park, Norwich, England; 2 Faculty of Applied Sciences, University of Sunderland, Sunderland, England; 3 Brewlab Ltd, Sunderland Enterprise Park, Sunderland, England; 4 Crisp Malting Group Ltd, Fakenham, Norfolk, England; Julius Kuhn-Institut, GERMANY

## Abstract

Wide crosses between genetically diverged parents may reveal novel loci for crop improvement that are not apparent in crosses between elite cultivars. The landrace Chevallier was a noted malting barley first grown in 1820. To identify potentially novel alleles for agronomic traits, Chevallier was crossed with the modern malting cultivar NFC Tipple generating two genetically diverse recombinant inbred line populations. Genetic maps were produced using genotyping-by-sequencing and 384-SNP genotyping, and the populations were phenotyped for agronomic traits to allow the identification of quantitative trait loci (QTL). Within the *semi-dwarf 1* (*sdw1*) region on chromosome 3H Chevallier conferred increased plant height and reduced tiller number, with QTL for these traits explaining 79.4% and 35.2% of the phenotypic variance observed, respectively. Chevallier was also associated with powdery mildew susceptibility, with a QTL on 1H accounting for up to 19.1% of the variance and resistance at this locus most likely resulting from an *Mla* variant from Tipple. Two novel QTL for physiological leaf spotting were identified on 3H and 7H, explaining up to 17.1% of the variance and with the Chevallier allele reducing symptom severity on 7H. Preliminary micromalting analysis was also undertaken to compare the malting characteristics of Chevallier and Tipple. Chevallier malt contained significantly lower levels of both α-amylase and wort β-glucan than Tipple malt, however no significant differences were observed for the remaining malting parameters measured. This suggests that the most obvious improvements in barley since the introduction of Chevallier are for agronomic traits such as height, yield and lodging resistance rather than for malting characteristics. Overall, our results demonstrate that this wide cross between Chevallier and Tipple may provide a source of novel QTL for barley breeding.

## Introduction

Two-row barleys, which are considered to produce premium malt for the beverage industry and have historically been preferred by European maltsters, are increasingly being favoured over six-row type in North America [1]. For continued improvement of two-row barleys, novel genes for agronomic, disease resistance and grain quality traits are needed. Chevallier is a two-row, English landrace barley which was first grown in Suffolk, England in 1820 [2]. During the nineteenth century Chevallier was widely regarded as one of the best malting cultivars in England, with Hallett’s Pedigree Chevallier being favoured by brewers until at least 1890 [3]. Due to its professed superior malt quality, good yield on chalky or sandy soils and the regularity of the shape, colour and size of its grain [4], up to 80% of the barley grown in England in the 1880s was derived from Chevallier [5]. However, Chevallier was also noted for its reduced tiller production and its propensity to lodge during wet seasons [6] and by 1920 very little Chevallier was being grown in England. Chevallier was selected prior to the advent of breeding for improved traits and thus represents a baseline for the impacts of barley improvement. Since the 1800s the desired characteristics of barley cultivars have greatly evolved, and it is unknown how the agronomic qualities of Chevallier compares to elite barley cultivars. There is much interest in increasing genetic diversity within modern barley germplasm and studies of landrace barleys have identified novel alleles associated with abiotic and biotic stresses [7, 8] and malting quality [9, 10]. It is therefore possible that Chevallier may also possess favourable characteristics or novel alleles that could be of potential use in modern breeding programmes.

Chevallier was recently revived from the Germplasm Resources Unit (GRU) at the John Innes Centre (JIC), Norwich, UK and was crossed with the modern malting cultivar NFC Tipple to produce two recombinant inbred line (RIL) populations with a diverse genetic background. NFC Tipple (hereafter termed ‘Tipple’) is a two-row, spring malting barley first released in 2004 (Syngenta Seeds, Ltd). Tipple has a shorter stem phenotype, resistance to both powdery mildew and brown rust, gained full Malting Barley Committee (MBC) approval for brewing use in 2007 and was on the AHDB Recommended List from 2005–2015 [11].

The aim of this study was to compare the agronomic characteristics of Chevallier and the elite cultivar Tipple under modern growing conditions and to identify any potential favourable alleles for barley improvement which originate from the Chevallier background. To achieve maximum resolution and reveal potentially novel genes as quantitative trait loci (QTL), we performed genetic mapping using the Chevallier × Tipple RIL populations, which were phenotyped for agronomically important traits such as tiller number, and powdery mildew and physiological leaf spotting resistance. To determine whether Chevallier also possessed any favourable quality traits micromalting analysis was also undertaken on Chevallier and Tipple malt, therefore providing the first preliminary analysis of Chevallier malt under contemporary malting conditions.

## Materials and methods

### Plant materials

Two separate bi-parental crosses were developed by single seed descent using Chevallier (JIC GRU accession 4851) and NFC Tipple (Syngenta Seeds, Ltd), which has the pedigree (NFC497-12 × Cork) × Vortex [12]. Two RIL populations were developed to determine whether phenotypic traits could be identified in similar genomic positions in both populations. An F_5_ population (C×T F_5_), with the last single seed selection made at the F_5_ generation was produced by KWS UK Ltd., Cambridge, UK and a second separate population, C×T F_7,_ was developed at JIC with the last single seed selection made at the F_7_ generation. The F_5_ population was used for preliminary mapping purposes, and the F_7_ population was used to give greater precision in the QTL mapping process due the more fixed genetic background. A total of 188 RILs were produced per population.

### Phenotyping

The populations were evaluated from 2013 to 2017 at JIC, Norwich, UK. All plots (1m^2^) were sown at a constant density and for all trials 12 randomised parental controls were included. The F_5_ population was trialled in 2013 in a whole plot single replicate trial, consisting of 10 field rows each containing 20 plots. Traits measured in this trial included plant height, tillering, physiological leaf spotting (PLS) and powdery mildew susceptibility. The F_7_ population was evaluated in a whole plot single replicate design in 2014, with 10 field rows each containing 20 plots, with height and mildew data recorded. Two further trials with the F_7_ population were sown in 2015 and 2017, using a split plot design, with each trial consisting of 10 field rows containing 40 split plots, with two replicates per line. Height, tillering, mildew susceptibility and PLS were scored. Tillering was scored on a 1–9 scale (1 = very poor, 9 = extensive tillering) from Zadoks GS21–29 [13]. PLS and mildew were scored from Zadoks GS14–39. PLS was observed as dark brown spots on both sides of the leaf and scored on a 1–10 scale (1 = no spotting, 10 = more than 50% of leaves covered). Mildew was scored on a 1–9 scale (1 = no visible symptoms, 9 = fully expanded leaves more than 50% infected). In all trials, the standard application of herbicides was used.

### Micromalting analysis

To provide preliminary data on malting quality, Chevallier, Tipple and a 105 subset of lines from the F_5_ population, based on seed availability, were sown at Morley Farm Ltd, Norfolk, UK in 2013 to provide seed for micromalting analysis. Two replicates of Chevallier and Tipple were sown, and a single replicate of each RIL was sown. Plots measured 2×6m, nitrogen was applied at 65kg/ha and the standard application of herbicides and fungicides for malting barley was used. Plots were harvested and graded over a 2.5mm sieve, with a 500g sample of seed selected for micromalting. Micromalting analysis was undertaken by Crisp Malting Group Ltd., Fakenham, England, using a standard steep and germination regime. The following malting quality traits were measured using the Institute of Brewing (IoB) standard recommended methods of analysis: α-amylase (dextrinizing units/du), diastatic power (°IoB and °WK), wort β-glucan (mg/l), IoB extract 0.7mm (L°/kg), free amino nitrogen (mg/l), soluble nitrogen ratio (%), total soluble nitrogen (%) and total nitrogen (%). Two replicates of the parental lines and a single replicate of the RILs were micromalted.

### Statistical analysis

Analyses of variance (ANOVA) for phenotypic traits were conducted separately for each environment by means of a general linear model (GLM) within Genstat 18^th^ edition (Lawes Agricultural Trust, Rothamsted Experimental Station, UK), to account for the effect of genotype, row (within the field) and replicate (for the 2015 and 2017 trials). Predicted means for each RIL were calculated within the GLM and t-probabilities were calculated to determine significant differences between the means of the parental lines and the RILs. For the micromalting data, two-sample t-tests were used to determine if the mean trait values of Chevallier and Tipple were significantly different.

### Genotyping and QTL analysis

#### 384-SNP genotyping

The F_5_ and F_7_ populations were genotyped using the 384-single nucleotide polymorphism (SNP) BeadXpress cultivar optimised genotyping panel, as described by Moragues et al. [14] and the genotyping-by-sequencing (GBS) method of Elshire et al. [15]. For the 384-SNP assay, leaf material from five 3-week old seedlings per line was pooled for the F_5_ population. As the population was developed to the F_5_ generation, the decision to pool the material was undertaken with the aim of accounting for any residual heterozygosity in individual seedlings. Genomic DNA was extracted using a Qiagen DNeasy 96 Plant Kit and genotyping using the 384-SNP assay was undertaken using the Illumina BeadXpress platform at the James Hutton Institute, Dundee, Scotland. SNP calls were analysed using Illumina BeadStudio software. From the SNP data the percentage heterozygosity in the population was as expected (6%). Due to the assumed lower heterozygosity in the F_7_ population, as this is a more genetically advanced population, leaf material from a single 3-week old seedling per line was sampled. After genotyping, the level of heterozygosity in the population was calculated to be as expected for an F_7_ population (<2%).

#### Genotyping by sequencing

As the heterozygosity in the F_5_ population was only 6%, leaf material from a single 3-week-old seedling per genotype was sampled for the F_5_ population for GBS genotyping. The same protocol was followed for the F_7_ population. Genomic DNA was extracted using a Qiagen DNeasy 96 Plant Kit. GBS libraries were produced at the Biotechnology Resource Centre Genomic Diversity Facility at Cornell University, New York, USA, using a 96-plex *Ape*KI restriction enzyme approach [15]. Libraries were sequenced using an Illumina HiSeq 2000/2500 generating 100bp single end reads. On average, 2,066,581 reads were produced for the C×T F_5_ population and 2,230,103 were produced for the F_7_ population per DNA sample. Raw sequence reads were trimmed and the barcodes removed, with the resulting 64bp tags mapped to the Morex reference genome to call the SNPs [16].

#### Genetic linkage mapping

For genetic mapping, SNPs which were monomorphic or displayed missing calls for either of the parental genotypes were removed from the analyses, as were SNPs with over 20% missing values for the C×T RILs. Individual marker chi-squared values were calculated and markers which deviated significantly from the expected 1:1 ratio were either re-checked if possible or removed from the marker set. Genetic maps were created from the combined datasets from both genotyping methods in JoinMap 3.0 [17] and genetic distances were calculated using the Kosambi mapping function, with a LOD threshold of 7.0 required to create linkage groups. The marker order of the linkage groups was referenced against both the barley consensus map and the Morex reference genome [16, 18], and incorrectly ordered or genetically redundant markers which mapped to the same genomic position within a linkage group were removed from the final maps.

#### QTL analysis

Genstat 18^th^ edition was used for QTL analysis, using predicted means generated within the GLM for each trait. Single-trait single environment analysis was performed on the F_5_ and F_7_ field trial and the F_5_ micromalting data, and single-trait multi-environment (ME) QTL analysis was used to determine genotype × environment interactions for the F_7_ dataset. For single environment QTL analysis, a LOD score of 3.0 was required to detect significant QTL and a maximum step size of 5cM was used. Simple interval mapping (SIM) was used for initial QTL detection, followed by two rounds of composite interval mapping (CIM) to finalise the QTL location using the detected candidate QTL as co-factors. A final QTL model was then fitted to produce the estimated QTL effects. For ME analysis the most appropriate variance-covariance matrix to model the correlations between the different environments was selected for each dataset, and SIM and CIM mapping was performed using the thresholds as described above. From the ME analysis, only QTL which did not display a genotype × environment interaction, and were therefore stable across environments, are presented. QTL names were assigned using the following nomenclature: “trait” + “population” + “year” + “chromosome”. ME QTL names were assigned as “trait” + “ME” + “chromosome”. QTL images were produced using MapChart [19].

## Results

### Phenotyping of Chevallier and Tipple

Chevallier was consistently tall in all trials, with the mean height ranging from 120.0cm in 2014 to 145.0cm in 2013 (Table 1). The mean height of Tipple ranged from 70.0cm in 2014 to 91.0cm in 2015. The differences in the predicted height between the two parents were significant in all datasets at the *P <*0.01 level (Table 1). Tipple had more tillers than Chevallier in all trials, with a mean score of 7.0 compared to 6.0 in Chevallier. However, the differences in predicted tillering scores were only significant in 2017 (*P <*0.05). Chevallier was more susceptible to powdery mildew than Tipple in all environments, with the predicted mean values between the parents being significantly different in all years (Table 1). Differences in PLS severity between Chevallier and Tipple parents were significant at the *P <*0.05 level in 2013 and 2015, whilst there was no difference in 2017.

**Table 1:** Predicted mean values from general linear modelling (GLM) of phenotypic traits for Chevallier and Tipple, and the range of predicted means of the Chevallier × Tipple F_5_ and F_7_ RILs.

			Parents			RILs	
Trait^a^	Population	Year	Chevallier	Tipple	t- probability^b^	Mean	Range
Height	F_5_	2013	145.0	79.0	0.002**	113.0	71.0–152.0
Height	F_7_	2014	120.0	70.0	<0.001***	95.0	61.0–124.0
Height	F_7_	2015	141.0	91.0	<0.001***	110.0	80.0–140.0
Height	F_7_	2017	124.0	71.0	<0.001***	94.0	61.0–127.0
Tillering	F_5_	2013	6.0	7.0	0.053	6.0	2.0–9.0
Tillering	F_7_	2015	6.0	7.0	0.061	6.0	3.0–8.0
Tillering	F_7_	2017	6.0	7.0	0.048*	5.0	3.0–8.0
Mildew	F_5_	2013	6.0	4.0	0.048*	5.0	2.0–9.0
Mildew	F_7_	2014	6.0	3.0	<0.001***	4.0	2.0–7.0
Mildew	F_7_	2015	5.0	4.0	0.041*	5.0	2.0–9.0
Mildew	F_7_	2017	5.0	3.0	0.020*	4.0	3.0–7.0
PLS	F_5_	2013	7.0	6.0	0.045*	6.0	1.0–10.0
PLS	F_7_	2015	4.0	5.0	0.042*	4.0	2.0–9.0
PLS	F_7_	2017	5.0	5.0	0.386	5.0	2.0–8.0

^a^ Height (cm), tillering/mildew (1–9 scale), PLS (1–10 scale).

^b^ The statistical significance of the difference between predicted mean scores for Chevallier and Tipple are shown by t-probabilities calculated within the GLM.

*, **, *** indicate *P* values of <0.05, <0.01 and <0.001, respectively.

### Phenotyping of Chevallier × Tipple RIL populations

The height for the RILs ranged from 71.0–152.0cm in the F_5_ population and 61.0–140.0cm in the F_7_ RILs (Table 1). T-probabilities were calculated to determine whether either the extreme short or tall RIL height values were significantly different to those of the appropriate parent and therefore an indicator of transgressive segregation. For height, the only significant difference was observed in 2015, with a difference between Tipple and the shortest F_7_ RIL (*P <*0.05). For tillering, the RIL scores ranged from 2.0–9.0 in the F_5_ population and 3.0–8.0 in the F_7_ population. Significant differences in tiller number between the extreme RILs and the parents were observed in all years: 2013 (*P <*0.05 and *P <*0.01 for Chevallier and Tipple respectively), 2015 (*P =* 0.001 and *P <*0.05 for Chevallier and Tipple respectively) and 2017 (*P <*0.01 for Chevallier), suggesting transgressive segregation for this trait. Mildew severity ranged from 2.0–9.0 in both the F_5_ and F_7_ RILs. Transgressive segregation for mildew resistance was observed in three years: 2013 and 2014 (*P <*0.05 for both Chevallier and Tipple), and 2015 (*P <*0.01 for both Chevallier and Tipple). PLS scores in the RILs ranged from 1.0–10.0 and 2.0–9.0 in the F_5_ and F_7_ populations, respectively (Table 1), with significant differences between Chevallier and Tipple and the most susceptible and resistant RILs being observed in all years: 2013 (*P <*0.01 and *P <*0.05 for Chevallier and Tipple respectively), 2015 (*P <*0.05 and *P <*0.01 for Chevallier and Tipple respectively) and 2017 (*P <*0.01 and *P =* 0.001 for Chevallier and Tipple respectively).

### Map construction

To create high density linkage groups for each chromosome, the SNPs identified from the 384-SNP assay were combined with those from the GBS method. A total of 172 markers from the 384-SNP assay were polymorphic between Chevallier and Tipple. Chromosomes 1H (14 SNPs) and 7H (11 SNPs) were particularly sparsely populated with polymorphic markers using this genotyping platform, whilst the greatest number of markers were identified on 3H (34 SNPs). With the GBS method 1577 filtered polymorphic SNPs were identified, with the fewest SNPs being identified on 4H (122 SNPs) and the greatest number being identified on 2H (205 SNPs). Data from the two genotyping methods was combined and genetically redundant markers which mapped to the same genomic position were removed. For each population a final combined map, with each chromosome represented by a single linkage group, was produced. The final F_5_ map contained 936 markers (95 384-SNP and 841 GBS markers) covering 1,224.4cM. The final combined F_7_ map contained 962 markers (135 384-SNP and 827 GBS markers), covering 1,078.4cM. A total of 857 SNP markers (90% of the total number of markers in each map) were identified as common markers between the F_5_ and F_7_ genetic maps. GBS reduces any potential effect of ascertainment bias as no prior knowledge of polymorphic loci is required to genotype a population [20], and by using this genotyping method approximately six times the number of polymorphic markers were identified between Chevallier and Tipple than were identified using the 384-SNP assay.

### Agronomic trait QTL analysis

A major QTL associated with height was identified on 3HL in both the F_5_ and F_7_ populations, in all trials. This QTL was located at 139.8cM in the F_5_ population, and at 135.5–136.5cM in the F_7_ population and accounted for up to 79.4% of the phenotypic variance (Table 2). This QTL was also identified from ME QTL analysis at the consensus position of 136.5cM in the F_7_ population, demonstrating that the QTL is stable across environments (Table 3). A minor height QTL was also identified on the short arm of 3H in all datasets. Again, this QTL was identified in the ME analysis of the F_7_ data, at 64.8cM, explaining up to 6.9% of the variance. Increased height at both QTL was associated with the Chevallier allele (Table 2). A major QTL for tillering was identified on 3HL in all trials (qT-F5-13.3H/ qT-F7-15.3H/ qT-F7-17.3H), explaining up to 31.7% of the phenotypic variance and with Tipple conferring the allele for increased tiller number (Table 2). This QTL was identified at the consensus position of 135.4cM in the F_7_ population by ME analysis, therefore co-locating with the major height QTL also on 3HL (Table 3). An additional tillering QTL (qT-F7-15.2H), was detected on 2H only in 2015, with the Chevallier allele conferring an increased tiller number. A major mildew QTL was identified on 1HS (qM-F7-15.1H/ qM-F7-17.1H), accounting for up to 19.1% of the phenotypic variance (Table 2). This QTL was identified at the consensus position of 19.3cM in the F_7_ population from the ME QTL analysis. The additional mildew QTL on 1H and 2H (qM-F5-13.1H, qM-F7-14.2H, qM-F7-15-.1H.2) were each only identified in a single trial year, with qM-F5-13.1H and qM-F7-15-.1H.2 mapping to a similar genomic region. A major PLS QTL was identified on 7HS in the F_7_ population at the consensus position of 55.0cM, explaining up to 17.1% of the variance observed and with the high allele originating from Tipple (Table 3). A second minor QTL on the long arm of 7H was identified in 2015, at 146.7cM, explaining up to 6.5% of the phenotypic variance. At this locus, Chevallier contributed the high value allele. QTL associated with PLS were also detected on 3H in the F_5_ and F_7_ populations each in a single trial year (qPLS-F5-13.3H, qPLS-F7-15.3H). All QTL positions are shown in S1–S7 Figs.

**Table 2:** Agronomic trait QTL identified from single-trait, single-environment QTL analysis in the Chevallier × Tipple F_5_ and F_7_ RILs.

QTL	Trait	Marker	Chr	Position	-LOG(P)	% Var	Add.	Allele	s.e.
qHT-F5-13.3H	Height	11_21197	3H	32.6	11.6	14.7	7.7	Chevallier	1.02
qHT-F7-14.3H	Height	44504	3H	66.6	9.3	4.3	3.7	Chevallier	0.56
qHT-F7-15.3H	Height	135476	3H	64.6	16.5	9.6	5.1	Chevallier	0.54
qHT-F7-17.3H	Height	11_10601	3H	60.7	5.1	3.2	3.2	Chevallier	0.68
qHT-F5-13.3H.2	Height	11_11172	3H	139.8	35.5	68.0	16.5	Chevallier	1.04
qHT-F7-14.3H.2	Height	45775	3H	136.5	67.9	79.4	15.9	Chevallier	0.56
qHT-F7-15.3H.2	Height	42877	3H	135.5	61.6	71.6	13.8	Chevallier	0.54
qHT-F7-17.3H.2	Height	45775	3H	136.5	53.7	73.3	15.2	Chevallier	0.68
qT-F5-13.3H	Tillering	11_11172	3H	139.8	12.1	31.7	0.9	Tipple	0.12
qT-F7-15.2H	Tillering	137043	2H	123.4	4.4	6.9	0.2	Chevallier	0.05
qT-F7-15.3H	Tillering	1594047	3H	135.4	12.6	24.0	0.4	Tipple	0.05
qT-F7-17.3H	Tillering	45775	3H	136.5	17.8	35.2	0.6	Tipple	0.06
qM-F5-13.1H	Mildew	11_10332	1H	43.9	3.9	9.6	0.4	Chevallier	0.10
qM-F7-14.2H	Mildew	66277	2H	129.6	4.1	8.4	0.3	Chevallier	0.09
qM-F7-15.1H	Mildew	42369	1H	18.3	10.3	19.1	0.5	Chevallier	0.07
qM-F7-15.1H.2	Mildew	139014	1H	63.0	4.8	7.9	0.3	Chevallier	0.07
qM-F7-17.1H	Mildew	183238	1H	21.0	4.7	9.4	0.3	Chevallier	0.06
qPLS-F5-13.3H	PLS	11_10312	3H	144.5	5.2	15.6	0.8	Chevallier	0.17
qPLS-F7.15.3H	PLS	1919082	3H	75.5	5.6	9.1	0.4	Chevallier	0.08
qPLS-F7.15.7H	PLS	435184	7H	55.0	9.3	17.1	0.6	Tipple	0.09
qPLS-F7.15.7H.2	PLS	41160	7H	146.7	4.0	6.5	0.4	Chevallier	0.08
qPLS-F7-17.7H	PLS	435184	7H	55.0	10.0	14.1	0.4	Tipple	0.08

**Table 3:** Stable agronomic trait QTL identified from single-trait, multiple-environment QTL analysis in the Chevallier × Tipple F_7_ RILs.

QTL^a^	Trait	Marker	Chr	Position	-LOG(P)	% Var	Add.	Allele	s.e.
qHT-ME.3H	Height	1572844	3H	64.8	16.8	6.9	4.1	Chevallier	0.48
qHT-ME.3H.2	Height	45775	3H	136.5	71.9	75.0	15.5	Chevallier	0.63
qT-ME.3H.2	Tillering	1594047	3H	135.4	36.9	40.6	0.5	Tipple	0.04
qM-ME.1H	Mildew	51610	1H	19.3	9.1	10.7	0.3	Chevallier	0.05
qPLS-ME.7H	PLS	435184	7H	55.0	15.1	20.4	0.5	Tipple	0.06

^a^ QTL are stable across environments and do not display a genotype × environment interaction.

### Malting quality analysis

Initial micromalting analysis was undertaken on Chevallier and Tipple malt to determine whether they differed for any malting quality parameters. Significant differences in micromalting quality between Chevallier and Tipple were observed for wort β-glucan and α-amylase (Table 4). Mean wort β-glucan values of 278.5 and 383.0 mg/l were observed for Chevallier and Tipple respectively, differences significant at the *P <*0.05 level (*P =* 0.036). The mean α-amylase values for Chevallier and Tipple were also significant at the *P <*0.05 level (*P =* 0.033), with values of 53.5 and 67.0 du, respectively (Table 4). However, there were no significant differences in the mean values of Chevallier and Tipple for the remaining traits: diastatic power, extract, free amino nitrogen, soluble nitrogen ratio, total soluble nitrogen and total nitrogen (Table 4).

**Table 4:** The mean values associated with malting traits for Chevallier and Tipple.

Trait ^a^	Chevallier	Tipple	t- probability^b^
α-amylase	53.5	67.0	0.033*
IoB diastatic power	106.0	147.0	0.148
Diastatic power	394.5	403.0	0.346
Wort β-glucan	278.5	383.0	0.036*
Extract	300.0	306.0	0.645
Free amino nitrogen	166.8	184.0	0.600
Soluble nitrogen ratio	36.7	40.2	0.671
Total nitrogen	1.8	1.9	0.836
Total soluble nitrogen	0.7	0.8	0.616

^a^ α-amylase: du; IoB diastatic power: °IoB; diastatic power: °WK; wort β-glucan: mg/l; extract: l°/kg; free amino nitrogen: mg/l; soluble nitrogen ratio: %; total nitrogen: % and total soluble nitrogen: %.

^b^ The statistical significance of the difference between mean values for Chevallier and Tipple was calculated from a t-test.

* indicates *P* value <0.05.

Preliminary micromalting was also undertaken for 105 lines of the F_5_ RIL population. The range of values for all analysed traits within the RIL population exceeded the values of the parental genotypes, suggesting the potential for transgressive segregation within the population (S1 Table). QTL analysis was performed using the preliminary data. QTL associated with malting parameters were identified on every chromosome except 1H, 5H and 6H (S2 Table) [21–29].

## Discussion

We developed an F_5_ Chevallier × Tipple population for agronomic trait identification, then used a separate Chevallier × Tipple cross that was progressed to the F_7_ generation to further confirm the position of several of these traits. We also combined two methods, a 384-SNP BeadXpress system and genotyping-by-sequencing, when genotyping both populations. This resulted in high density genetic linkage maps with good marker coverage, which revealed potentially new QTL. The genetic maps generated from combining the SNPs identified from these two genotyping methods covered 1,224.4cM and 1,078.4cM in the F_5_ and F_7_ populations, respectively, distances which are comparable to those in other barley studies generated using similar genotyping methods [18, 30, 31].

### Agronomic trait QTL

Tipple has been shown to possess the *sdw1*.*d* allele of the semi-dwarf 1 (*sdw1*) gene, which is widely used in European barley breeding programmes [32]. *sdw1*.*d* is located at the distal end of 3HL and is associated with a reduction in plant height and thousand-grain weight, late heading and increased tiller number [33]. The presence of the *sdw1*.*d* allele in Tipple largely explains the differences in height and tiller number between the two parents, as demonstrated by the major QTL on 3HL in both of the C×T populations. However, the analysis revealed that Chevallier also carries a second QTL on 3HS for increased plant height, in a similar location as identified in other studies [34, 35], though this had a lesser but consistent effect.

Our analysis reveals novel information about the genetics of quantitative disease resistance (QDR) to powdery mildew in barley. We defined three QTL for powdery mildew susceptibility in the Chevallier background dispersed on chromosomes 1H and 2H, with contributions to variance ranging from 7.9% to 19.1%. Likely interactions between these QTL are evidenced by transgressive segregation for the trait, with some progeny lines comparatively resistant (score 2) compared to the Chevallier or Tipple parents (Table 1). Our results also demonstrate that the source of powdery mildew resistance in Tipple cannot be *mlo*. *Mlo* is located on chromosome 4H and provides durable, broad-spectrum resistance to all races of powdery mildew [36]. However, *mlo* is also associated with a yield penalty, physiological leaf spotting (PLS) and can increase susceptibility to the hemibiotrophic pathogen *Ramularia collo-cygni* which causes Ramularia leaf spot [37, 38]. Whilst Tipple has good mildew resistance and there have been no reports of a resistance breakdown, its *mlo* status has remained unclear. In Europe, *mlo-11* is the most widely used allele in spring barley breeding programmes [39]. However, our investigation revealed that no QTL associated with mildew were observed on 4H and so *mlo* cannot be the source of resistance. Our conclusion that Tipple does not possess *mlo-11* is supported by results from the United Kingdom Cereal Pathogen Virulence Survey (UKCPVS) from 2012 to 2017 [40] comparing Tipple and the *mlo-11* carrying cultivars Apex and Riviera. Chevallier is also unlikely to possesses any form of *mlo*-associated resistance because it was selected prior to the advent of modern breeding programmes and is very susceptible to powdery mildew. The positioning of the major QTL for powdery mildew resistance on 1H suggests it is highly likely to be *Mla* from the Tipple background. Several differential lines containing *Mla* resistance (*Mla1*, *Mla3*, *Mla6*, *Mla7*, *Mla9*, *Mla12* and *Mla13*) are included in the UKCPVS panel, with the resistance profile of Tipple being most like that of Ricardo, a cultivar which possesses *Mla3* [40]. It is possible that Tipple may therefore also carry *Mla3*, a resistance gene for which virulent mildew isolates appear to have been present at a low frequency in the UK pathogen population between 2012–2017 [40]. The low frequency of *Mla3*-virulent isolates could also explain why the 1H QTL, at the consensus position of 19.3cM, only accounts for up to 19.1% of the variance.

Physiological leaf spotting (PLS) is an abiotic foliar disease of barley, which affects the capacity for grain filling and can reduce yield by up to 20% [41]. The characteristic symptoms of PLS are similar to those of Ramularia leaf spot and net blotch, caused by *Pyrenophora teres* f. *teres* [41], meaning the presence of PLS can make it difficult to accurately score biotic foliar diseases. Our investigation revealed novel QTL for PLS, and also confirmed those previously reported. Increased susceptibility to abiotic leaf spotting is often associated with the presence of *mlo* alleles conferring powdery mildew resistance [37]. None of the PLS QTL were located on 4H, again supporting our conclusion that *mlo* is not present within the population. In our study, PLS QTL were located on 3H and 7H. Both parental lines contribute towards PLS severity which also explains the transgressive segregation for the trait seen within the RILs. QTL on 3H associated with PLS have not been previously reported, suggesting that qPLS-F5-13.3H/qPLS-F7-15.3H may be novel. A major PLS QTL, qPLS-ME.7H, accounting for up to 20.4% of the phenotypic variance, was detected at the same marker on 7HS in the F_7_ population in 2015 and 2017. Increased severity at this location was associated with the Tipple allele. Behn et al. [42] identified a minor PLS QTL on 7HS in a PZ24727 × Barke population, which explained 3.4% of the phenotypic variance. In that study, however, the reported QTL was also coincident with mildew susceptibility, an association not seen in our C×T population. This suggests that the QTL on 7H in the C×T background, with reduced severity associated with the Chevallier allele, is also novel.

### Micromalting

In commercial production of malting barley, the type of soil and fertilizer regime has a major impact on malt quality parameters. Although our trial plots were not optimised for large scale malting barley production, we were able to make initial comparisons between the malting characteristics of Chevallier and Tipple using replicated micromalting samples. Moreover, our preliminary mapping of malting parameters in the C×T F_5_ population revealed previously-identified QTL as well as potentially novel QTL for some traits (S2 Table). More robust mapping of these traits in the C×T cross could be achieved with repeated experiments in different environments over multiple years.

Surprisingly, our investigation revealed that the malting quality parameters of Chevallier compare favourably to those of Tipple, a recently-introduced elite malting barley cultivar. β-glucan content of malt provides an indication of the extent of endosperm breakdown, and a preferred wort β-glucan content should ideally be less than 200mg/l [43] to provide sufficient modification without increased wort viscosity. Although both cultivars exceed this value, the wort β-glucan level of Chevallier (278.5 mg/l) is significantly lower than Tipple (383.0 mg/l). Diastatic power is a measure of the combined enzyme activity of α-amylase and the additional diastatic enzymes β-amylase and limit dextrinase [44]. The mean diastatic power values for both Chevallier and Tipple were 394.5 and 403.0°WK respectively, values which were not significantly different and therefore indicate similar levels of starch degradation. Chevallier malt has a significantly lower α-amylase content than Tipple, but a comparable overall diastatic power content. This suggests that Chevallier malt may contain higher levels of β-amylase and limit dextrinase than α-amylase, however additional analysis of these individual parameters would be required to confirm this. FAN provides an essential source of yeast nutrition and therefore has an important impact on flavour and processing of beer [45]. A FAN content of 140 – 190mg/l within malt is desirable [46], meaning the predicted mean values for both Chevallier and Tipple are within the range required for adequate yeast fermentation. A total grain nitrogen (TN) content of 1.60–1.75% is preferred by most UK brewers [47], meaning that the TN content of both Chevallier and Tipple, at 1.8 and 1.9% respectively, somewhat exceed this value.

Overall, our investigation has revealed potentially novel loci associated with reduced physiological leaf spotting, QDR for powdery mildew and also favourable malt quality traits that could have potential benefit in barley breeding programmes and so warrant further investigation. Additionally, we show that Chevallier is capable of producing malt to acceptable modern standards which is remarkable considering that this landrace was first selected in the 1820s. This result suggests that the most obvious improvements in barley since the introduction of Chevallier are for agronomic traits such as height, yield and lodging resistance rather than for malting characteristics. With increasing interest in flavour attributes of malt [48], studies based on wide crosses such as this Chevallier × Tipple population could reveal novel loci for future barley improvement and selection.

## Supporting information

S1 Fig QTL identified on chromosome 1H. a) QTL in the Chevallier × Tipple F_5_ population and b) QTL in the Chevallier × Tipple F_7_ population.
(PDF)

S2 Fig QTL identified on chromosome 2H. a) QTL in the Chevallier × Tipple F_5_ population and b) QTL in the Chevallier × Tipple F_7_ population.
(PDF)

S3 Fig QTL identified on chromosome 3H. a) QTL in the Chevallier × Tipple F_5_ population and b) QTL in the Chevallier × Tipple F_7_ population.
(PDF)

S4 Fig QTL identified on chromosome 4H. a) QTL in the Chevallier × Tipple F_5_ population and b) QTL in the Chevallier × Tipple F_7_ population.
(PDF)

S5 Fig QTL identified on chromosome 5H. a) QTL in the Chevallier × Tipple F_5_ population and b) QTL in the Chevallier × Tipple F_7_ population.
(PDF)

S6 Fig QTL identified on chromosome 6H. a) QTL in the Chevallier × Tipple F_5_ population and b) QTL in the Chevallier × Tipple F_7_ population.
(PDF)

S7 Fig QTL identified on chromosome 7H. a) QTL in the Chevallier × Tipple F_5_ population and b) QTL in the Chevallier × Tipple F_7_ population.
(PDF)

S1 Table The range and mean micromalting values of 105 Chevallier ×Tipple F_5_ RILs. (PDF)

S2 Table Malting trait QTL identified from 105 Chevallier ×Tipple F_5_ RILs. (PDF)
