# Supplementary material for: Mapping of agronomic traits, disease resistance and malting quality in a wide cross of two-row barley cultivars
Source: PLoS One. 2019 Jul 17;14(7):e0219042. doi: 10.1371/journal.pone.0219042 (PMC6636724; doi:10.1371/journal.pone.0219042)
Supplement: S3 Fig — a) QTL in the Chevallier × Tipple F5 population and b) QTL in the Chevallier × Tipple F7 population. (PDF) [file pone.0219042.s003.pdf]

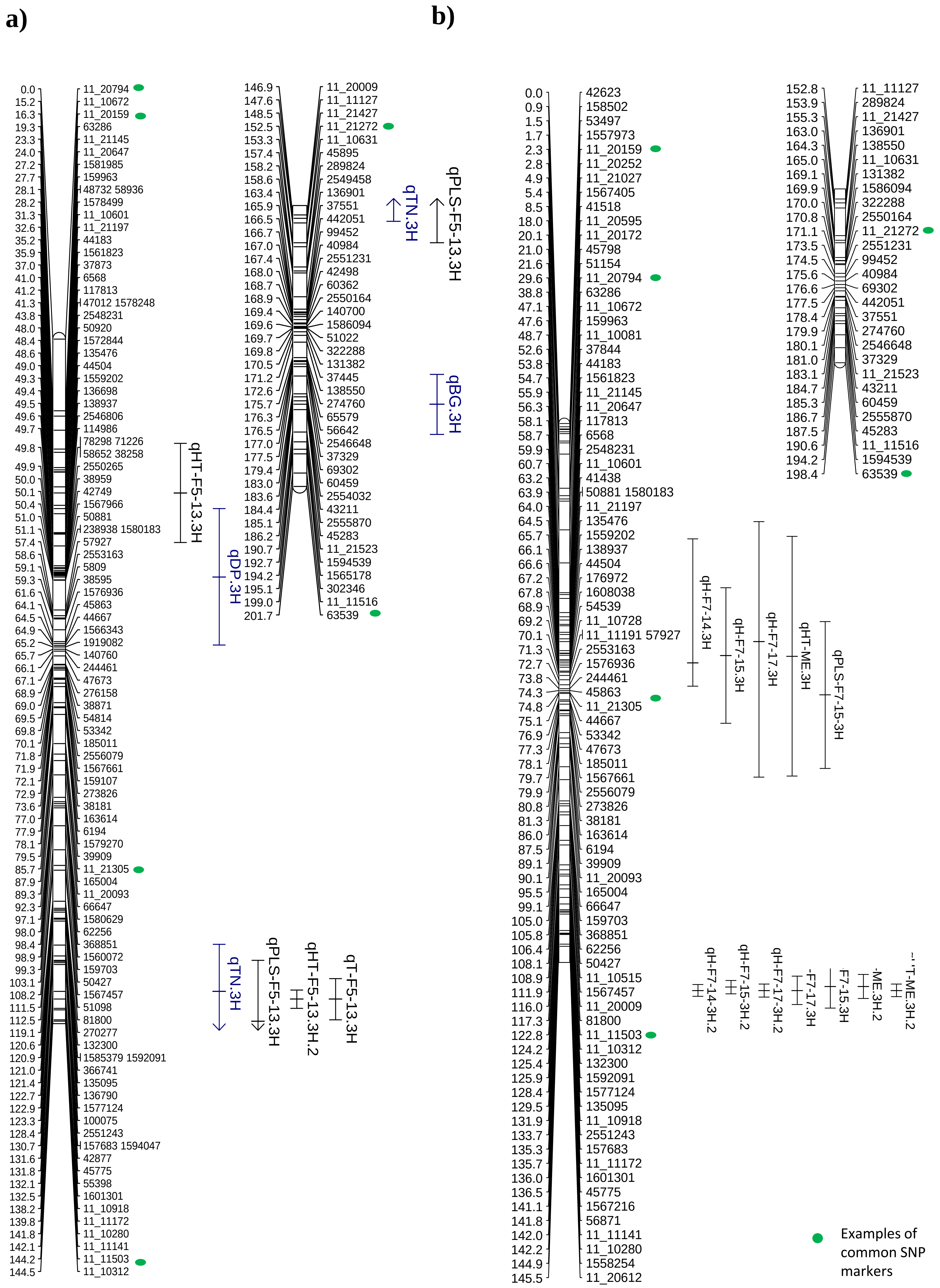

S3 Fig. QTL identified on chromosome 3H. a) QTL in the Chevallier × Tipple F5 population and b) QTL in the Chevallier × Tipple F7 population.
