# Supplementary material for: Mapping of agronomic traits, disease resistance and malting quality in a wide cross of two-row barley cultivars
Source: PLoS One. 2019 Jul 17;14(7):e0219042. doi: 10.1371/journal.pone.0219042 (PMC6636724; doi:10.1371/journal.pone.0219042)
Supplement: S1 Table — (PDF) [file pone.0219042.s008.pdf]

S1 Table. The range and mean micromalting values of 105 Chevallier ×Tipple F_5 RILs.

Trait [a]	Mean	Range
α-amylase	69.4	44.0 – 146.0
IoB diastatic power	188.8	58.0 – 342.0
Diastatic power	595.3	183.0 – 1077.0
Wort β-glucan	228.1	103.0 – 894.0
Extract	296.8	279.0 – 318.0
Free amino nitrogen	147.7	103.0 – 208.0
Soluble nitrogen ratio	35.7	28.8 – 45.6
Total nitrogen	1.9	1.7 – 2.4
Total soluble nitrogen	0.7	0.5– 0.9

[a] α-amylase: du; IoB diastatic power: °IoB; diastatic power: °WK; wort β-glucan: mg/l; extract: L°/kg; free amino nitrogen: mg/l; soluble nitrogen ratio: %; total nitrogen: % and total soluble nitrogen: %.
