# Supplementary material for: Mapping of agronomic traits, disease resistance and malting quality in a wide cross of two-row barley cultivars
Source: PLoS One. 2019 Jul 17;14(7):e0219042. doi: 10.1371/journal.pone.0219042 (PMC6636724; doi:10.1371/journal.pone.0219042)
Supplement: S2 Table — (PDF) [file pone.0219042.s009.pdf]

S2 Table. Malting trait QTL identified from 105 Chevallier ×Tipple F5 RILs.

QTL	Trait[a]	Marker	Chr	Position	-LOG(P)	% Var	Add.	Allele	s.e.	Reference[b]
qAA.7H	α-amylase	1561200	7H	162.9	3.9	16.0	7.9	Tipple	1.97	[21-23]
qDP.3H	Diastatic power	1567966	3H	50.4	3.0	11.4	52.7	Chevallier	15.87	
qBG.2H	Wort β-glucan	1769678	2H	214.9	3.6	14.4	64.7	Tipple	16.88	[22, 24, 25]
qBG.3H	Wort β-glucan	2555870	3H	185.1	5.3	23.3	82.2	Tipple	16.74	
qEX.2H	Extract	368439	2H	56.5	3.3	13.6	1.8	Tipple	0.50	[24, 26, 27]
qFAN.2H	Free amino nitrogen	106745	2H	207.4	3.6	10.6	6.8	Chevallier	1.98	
qFAN.4H	Free amino nitrogen	49161	4H	58.9	3.2	6.4	5.3	Chevallier	2.06	[27-29]
qFAN.4H.2	Free amino nitrogen	11_21490	4H	90.5	3.3	8.4	6.1	Chevallier	2.28	[27-29]
qSNR.2H	Soluble nitrogen ratio	374975	2H	206.4	3.4	8.2	0.9	Chevallier	0.32	[27]
qSNR.4H	Soluble nitrogen ratio	11_20012	4H	91.9	4.2	21.3	1.5	Chevallier	0.36	[27-29]
qTSN.4H	Total soluble nitrogen	11_20012	4H	91.9	3.3	17.8	0.1	Chevallier	0.01	[27]
qTN.3H	Total nitrogen	11_10918	3H	138.2	3.1	15.2	0.0	Chevallier	0.02	[27-29]

[a] α-amylase: du; diastatic power: °WK; wort β-glucan: mg/l; extract: L°/kg; free amino nitrogen: mg/l; soluble nitrogen ratio: %; total nitrogen: % and total soluble nitrogen: %.

[b] Reference for QTL previously identified within the literature.
